# The Number of Concomitant Drugs and the Safety of Direct Oral Anticoagulants in Routine Care Patients with Atrial Fibrillation

**DOI:** 10.1055/s-0040-1721499

**Published:** 2020-12-23

**Authors:** Carline J. van den Dries, Sander van Doorn, Patrick Souverein, Romin Pajouheshnia, Karel G.M. Moons, Arno W. Hoes, Geert-Jan Geersing, Hendrika A. van den Ham

**Affiliations:** 1 Julius Center for Health Sciences and Primary Care, University Medical Center Utrecht and Utrecht University, Utrecht, The Netherlands; 2 Division of Pharmacoepidemiology and Clinical Pharmacology, Utrecht Institute for Pharmaceutical Sciences, Utrecht University, Utrecht, The Netherlands

**Keywords:** atrial fibrillation, anticoagulation, polypharmacy, major bleeding, effect modification

## Abstract

**Background** The benefit of direct oral anticoagulants (DOACs) versus vitamin K antagonists (VKAs) on major bleeding was less prominent among atrial fibrillation (AF) patients with polypharmacy in post-hoc randomized controlled trials analyses. Whether this phenomenon also exists in routine care is unknown. The aim of the study is to investigate whether the number of concomitant drugs prescribed modifies safety and effectiveness of DOACs compared with VKAs in AF patients treated in general practice.

**Study Design** Adult, nonvalvular AF patients with a first DOAC or VKA prescription between January 2010 and July 2018 were included, using data from the United Kingdom Clinical Practice Research Datalink. Primary outcome was major bleeding, secondary outcomes included types of major bleeding, nonmajor bleeding, ischemic stroke, and all-cause mortality. Effect modification was assessed using Cox proportional hazard regression, stratified for the number of concomitant drugs into three strata (0–5, 6–8, ≥9 drugs), and by including the continuous variable in an interaction term with the exposure (DOAC vs. VKA).

**Results** A total of 63,600 patients with 146,059 person-years of follow-up were analyzed (39,840 person-years of DOAC follow-up). The median age was 76 years in both groups, the median number of concomitant drugs prescribed was 7. Overall, the hazard of major bleeding was similar between VKA-users and DOAC-users (hazard ratio [HR] 0.98; 95% confidence interval [CI] 0.87–1.11), though for apixaban a reduction in major bleeding was observed (HR 0.81; 95% CI 0.68–0.98). Risk of stroke was comparable, while risk of nonmajor bleeding was lower in DOAC users compared with VKA users (HR 0.92; 95% CI 0.88–0.97). We did not observe any evidence for an impact of polypharmacy on the relative risk of major bleeding between VKA and DOAC across our predefined three strata of concomitant drug use ( *p* -value for interaction = 0.65). For mortality, however, risk of mortality was highest among DOAC users, increasing with polypharmacy and independent of the type of DOAC prescribed ( *p* -value for interaction <0.01).

**Conclusion** In this large observational, population-wide study of AF patients, risk of bleeding, and ischemic stroke were comparable between DOACs and VKAs, irrespective of the number of concomitant drugs prescribed. In AF patients with increasing polypharmacy, our data appeared to suggest an unexplained yet increased risk of mortality in DOAC-treated patients, compared with VKA recipients.

## Introduction

In atrial fibrillation (AF) management, stroke prevention with anticoagulation is pivotal, as the risk of stroke is increased fivefold in AF patients if left untreated. 1 For many years, vitamin K antagonists (VKAs) have been the cornerstone therapy in anticoagulation management. Recently, direct acting oral anticoagulants (DOACs), also known as nonvitamin K oral anticoagulants (OACs), became the preferred alternative. 2 3 The original randomized trials on dabigatran, apixaban, rivaroxaban, and edoxaban all demonstrate that these drugs are as effective in reducing stroke risk compared with VKAs, while their risk of gastrointestinal bleeding is increased (except for apixaban) and their risk of major bleeding and especially intracranial hemorrhage decreased. 4

Patients with AF often use multiple drugs, as most AF patients are of high age and suffer from multiple comorbidities. 5 6 In two trials, post-hoc analyses examined the impact of polypharmacy (defined as ≥5 concomitant drugs) on the relative risk estimates of the DOACs rivaroxaban and apixaban versus warfarin on major bleeding, respectively. For both DOACs the risk of bleeding increased when the number of concomitant drugs prescribed increased. For apixaban versus warfarin, the benefits of bleeding risk reduction decreased when the number of drugs increased. 7 For rivaroxaban, the reduced risk of major bleeding as compared with warfarin even completely disappeared in patients using five or more drugs, which was also shown in a systematic review of these DOAC trials. 8 9 Whether these trial results are generalizable to patients with AF treated in routine care is debatable. The proportion of eligible patients who actually participates in randomized trials is often low (or unknown, as in the DOAC trials) and more importantly, characteristics of patients included in the trials often differ from the characteristics of patients treated in routine care. 10 11 This makes the generalizability of trial data particularly questionable in elderly patients, to whom DOACs nowadays are also increasingly prescribed. As the use of multiple concomitant drugs is generally the rule rather than the exception in the elderly 12 and because the number of concomitant drugs is easy to assess by clinicians, it would be valuable to know whether the number of concomitant drugs affects the safety and efficacy of DOACs compared with VKA in routine care, and, thus, whether this should be taken into account when choosing either treatment strategy over the other.

With this study, we aim to investigate whether the relative safety and efficacy of DOACs compared with VKAs are influenced by the number of concomitant drugs prescribed to AF-patients treated in routine practice. The United Kingdom Clinical Practice Research Datalink (CPRD) offers a unique opportunity to quantify the influence of the number of concomitant drugs prescribed on safety and efficacy outcomes in a large number of AF-patients in daily practice, followed over a long period of time.

## Materials and Methods

### Study Design and Data Source

This retrospective cohort study was performed using data from the United Kingdom CPRD. This large, widely used, and nationally representative dataset includes electronic health care records from over 11.3 million patients (covering 6.9% of the UK population) treated in general practice in the United Kingdom. 13 Available data from routine clinical practice include demographic characteristics, medical history, drug prescriptions, clinical events, and hospital referrals. Drug prescriptions are coded using the British National Formulary (BNF) and clinical symptoms and diagnoses are recorded with Read codes. The validity of the diagnoses recorded in CPRD was demonstrated in previous studies. 14 15 The study protocol was approved by the CPRD ISAC Committee (ISAC protocol number 18_241R). This manuscript was written in accordance with the STROBE guideline for reporting observational studies. 16

### Study Population

**Fig. 1:**
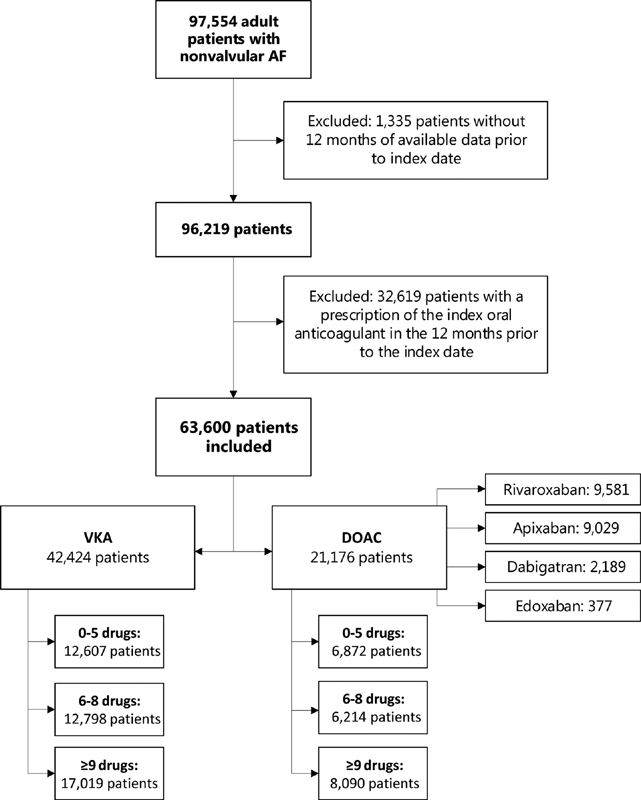
Flowchart showing selection of the study population.

Fig. 1 shows the selection of the study population. Adult patients with a first prescription of a DOAC or VKA during the period of January 1, 2010 to July 1, 2018 were included. To ensure that only *new* users were included, patients could not have a prescription of the same OAC in the 12 months prior to the date of the first prescription (index date). However, patients were not necessarily OAC naive: the group of patients with a first DOAC prescription could also include patients with previous VKA use (i.e., switchers), and vice versa.

Only patients with the diagnosis of AF, recorded ever before the index date, were included. To study patients with nonvalvular AF only, we excluded patients with a prosthetic heart valve or a history of rheumatic mitral valve stenosis. Patients needed to be enrolled in the database at least 12 months prior to the index date to ensure that valid baseline data were available. Follow-up ended when a patient had the outcome of interest or when a patient was censored (in case of death, moving out of the CPRD practice, end of data collection of the CPRD practice, or end of study period), or on the last day valid data were available (whichever occurred first). A separate dataset was created for each outcome.

### Exposure

Treatment episodes, defined as series of subsequent OAC prescriptions independent of dose changes, were constructed according to the method of Gardarsdottir et al, to define current use and past use of OACs. 17 A permissible gap time, or grace period, of 60 days between the theoretical end date of a prescription and the next prescription was allowed for, as patients may have had tablets left due to nonadherence or temporary discontinuation around invasive medical procedures or, in VKA users, in case of too high international normalized ratio (INR) values.

During the analysis phase, it appeared that a considerable number of major bleeding events occurred shortly after the end of a current use period. In fact, the incidence rate for major bleeding was higher in the period immediately following apparent discontinuation than during exposure to VKA or DOAC, which is highly improbable and is most likely explained by exposure misclassification at the time of the recorded outcome. If this follow-up time would indeed be classified as nonexposed, a third of all bleeding events would have been ignored. Moreover, this would have introduced a major source of bias, as for almost all “unexposed” periods the last anticoagulant used was a VKA, which seems reasonable given that VKAs are more prone to stockpiling than DOACs due to the varying dosage regimen. Therefore, we post-hoc reclassified the first treatment period (maximum 91 days) after apparent discontinuation to the last anticoagulant used for all analyses (i.e., a “last measurement carried forward” approach).

### Outcome

The primary outcome was major bleeding, defined as a symptomatic bleeding in one of the following critical areas or organs: intracranial, intraspinal, retroperitoneal, intraocular, gastrointestinal, intra-articular, or intrathoracic. This definition was chosen, as the definition of major bleeding recommended by the International Society on Thrombosis and Hemostasis (ISTH) 18 is difficult to use because of missing information about hemoglobin levels or blood transfusions in CPRD data.

Secondary outcomes were ischemic stroke, gastrointestinal bleeding, nonmajor bleeding, and all-cause mortality. Ischemic strokes registered during the first month of DOAC or VKA use were excluded, (i.e., a so called blanking or quarantine period), because in those cases an ischemic stroke is probably the first presentation of AF, when the anticoagulant had not yet been started. 19 Thus, in those cases the OAC is initiated because of the ischemic stroke and subsequent detection of AF, rather than the occurrence of an ischemic stroke during follow-up. Due to the possibility of late registration of the stroke in the GP registry, counting these strokes as outcome events during anticoagulation treatment would induce misclassification. 19 Lists of the Read codes defining each outcome are provided in the Supplementary Material (Section 1) .

### Effect Modification

**Fig. 2:**
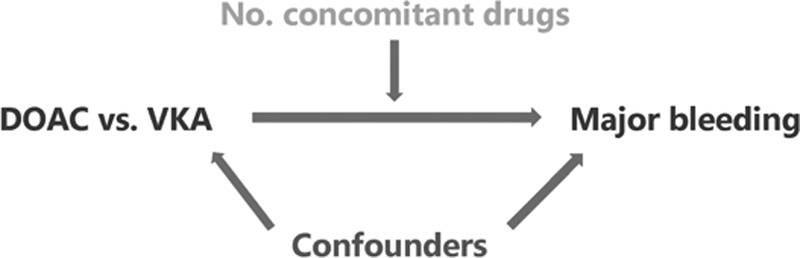
The relation between the exposure, primary outcome, effect modifier (the number of concomitant drugs, primarily of interest in this study), and confounders.

Fig. 2 shows a graphical display of the relations between the different variables in this study. The primary interest of this study was to quantify the influence of the number of concomitant drugs prescribed on the safety and efficacy of DOACs versus VKAs; thus, to quantify effect modification by the number of concomitant drugs. This variable was constructed by counting the total number of unique BNF codes prescribed to each patient during each treatment period, excluding all nonpharmacological prescriptions (for instance wound care bandages, stockings, stoma/incontinence materials).

### Confounding

A priori, we identified a separate set of possible confounders for each outcome based on prior evidence. For the primary outcome major bleeding, the following 17 patient characteristics were included as possible confounders in the analyses: age, sex, previous use of a different anticoagulant, alcohol abuse, liver disease, chronic kidney disease, hypertension (treated or untreated), history of gastrointestinal bleeding, history of intracranial bleeding, cardiovascular disease (defined as a history of ischemic heart disease, peripheral artery disease, stroke, or transient ischemic attack [TIA]), active cancer, peptic ulcer disease, concomitant use of platelet inhibitors, nonsteroidal anti-inflammatory drugs (NSAIDs), oral corticosteroids, proton pump inhibitors, or selective serotonin reuptake inhibitors. The confounders for the secondary outcomes are listed in the Supplementary Material (Section 2) . None of the confounding variables were possible intermediate variables in the relation between the exposure and the outcome.

### Statistical Analysis

All variables regarding exposure, confounding (except for sex and alcohol abuse), and the number of concomitant drugs prescribed were treated as time-varying variables and updated either when the exposure status changed, or every 90 days if the exposure remained unchanged. Incidence rates were reported as the number of events per 100 person-years. Cox proportional hazard regression models were used to estimate hazard ratios and their 95% confidence intervals when comparing DOACs with VKA. The proportional hazards assumption was assessed visually by plotting the scaled Schoenfeld residuals. 20 We used multivariable Cox regression to adjust for potential confounders mentioned above. To address the effect of the total number of concomitant drugs prescribed and answer our primary research question, we created three strata of the number of concomitant drugs prescribed: 0 to 5, 6 to 8, and 9 or more drugs. These cut-offs were chosen as they provided the most equal distribution of the number of patients across the strata. Next, to test for statistically significant effect modification, we included the continuous variable “number of concomitant drugs × OAC treatment” as an interaction term in the multivariate Cox regression model to derive the *p* -value for interaction. In case of few events compared with the number of confounding variables adjusted for, Firths correction (a penalized regression technique), with Wald confidence intervals and *p* -values, was applied to mitigate possible small sample bias. 21 Additionally, we investigated whether the results for major bleeding differed when separately comparing apixaban, rivaroxaban, and dabigatran to warfarin.

We performed four sensitivity analyses for the primary outcome (major bleeding). First, we analyzed the data without reclassifying any unexposed periods, so without our post-hoc defined “last measurement carried forward” approach. Second, we excluded patients who had other indications for OAC (for instance pulmonary embolism or knee/hip replacement surgery) registered within 3 months before and after the index date to ensure that AF was indeed the reason the anticoagulant was started. Third, we excluded prescriptions from the variable “number of concomitant drugs prescribed” which we regarded to be less relevant (first all topical drugs and second all incidental prescriptions, see Supplementary Material (Section 3) , for an overview of the BNF chapters that were excluded in the sensitivity analyses). Finally, we reclassified the strata of number of concomitant drugs used to 0 to 5 drugs, 6 to 9 drugs, and 10 or more drugs used.

A *p* -value of 0.05 or lower (or a 95% confidence interval not including a hazard ratio of 1) was considered statistically significant. All analyses were performed using *R* version 3.4.4 and *R* Studio version 1.1.442. 22 The package “survival” (version 2.38) was used for all Cox models, and the package “coxphf” (version 1.13) for Firths correction. 23 24

## Results

### Baseline Characteristics

In total, 63,600 AF patients were included (67% of patients using a VKA and 33% using a DOAC at cohort entry), contributing to a total of 146,059 person-years of follow-up for the primary outcome major bleeding. Median follow-up time was 2.0 years for VKA patients and 1.1 years for DOAC patients. Patients were exposed to a VKA during 106,219 person-years of follow-up (73%) and to a DOAC during 39,840 person-years of follow-up (27%). Rivaroxaban accounted for 48% of follow-up time exposed to DOAC, apixaban for 38%, dabigatran for 13%, and edoxaban for 4%. Baseline characteristics per stratum are shown in Table 1 . At baseline, 19,479 patients (31%) used zero to five concomitant drugs, 19,012 patients (30%) used six to eight concomitant drugs, and 25,019 patients (39%) used nine or more concomitant drugs.

**Table 1:** Baseline characteristics per stratum of the number of concomitant drugs prescribed

	Stratum 1 (0–5 drugs)		Stratum 2 (6–8 drugs)		Stratum 3 (≥9 drugs)	
	VKA ( *n* = 12,607)	DOAC ( *n* = 6,872)	VKA ( *n* = 12,798)	DOAC ( *n* = 6,214)	VKA ( *n* = 17,019)	DOAC ( *n* = 8,090)
Female	4,715 (37.4)	2,639 (38.4)	5,654 (44.2)	2,700 (43.5)	8,204 (48.2)	4,099 (50.7)
Age, median (IQR)	73 (65–80)	72 (65–80)	76 (69–82)	76 (69–84)	77 (71–83)	79 (72–85)
N. conc. drugs, median (IQR)	4 (3–5)	4 (3–5)	7 (6–8)	7 (6–8)	11 (10–14)	11 (10–14)
Previous use of different OAC	1 (0.0)	312 (4.5)	2 (0.0)	315 (5.1)	2 (0.0)	495 (6.1)
**Comorbidities/risk factors**						
Hypertension	5,806 (46.1)	3,102 (45.1)	8,604 (67.2)	4,166 (67.0)	12,585 (73.9)	5,890 (72.8)
Heart failure	813 (6.4)	365 (5.3)	1591 (12.4)	661 (10.6)	3,322 (19.5)	1,462 (18.1)
Diabetes	804 (6.4)	475 (6.9)	1833 (14.3)	1,038 (16.7)	5,152 (30.3)	2,449 (30.3)
Prior TIA or ischemic stroke	1,661 (13.2)	957 (13.9)	2,413 (18.9)	1,224 (19.7)	3,547 (20.8)	1,889 (23.3)
Prior VTE	509 (4.0)	145 (2.1)	534 (4.2)	163 (2.6)	953 (5.6)	323 (4.0)
Coronary artery disease	1,408 (11.2)	733 (10.7)	3,012 (23.5)	1,415 (22.8)	6,744 (39.6)	2,854 (35.3)
Presence of malignancy	403 (3.2)	217 (3.2)	426 (3.3)	244 (3.9)	730 (4.3)	334 (4.1)
Chronic kidney disease	1,662 (13.2)	826 (12.0)	2,914 (22.8)	1,320 (21.2)	5,416 (31.8)	2,478 (30.6)
Prior major bleeding	344 (2.7)	226 (3.3)	548 (4.3)	302 (4.9)	1,083 (6.4)	545 (6.7)
Peptic ulcer disease	534 (4.2)	289 (4.2)	807 (6.3)	409 (6.6)	1,432 (8.4)	712 (8.8)
Alcohol abuse	847 (6.7)	667 (9.7)	942 (7.4)	663 (10.7)	1,387 (8.1)	921 (11.4)
Active smoking	1,070 (8.5)	605 (8.8)	1,040 (8.1)	550 (8.9)	1,475 (8.7)	814 (10.1)
**Prior use of drugs affecting bleeding risk**						
Antiplatelet therapy	5,937 (47.1)	2,341 (34.1)	7,745 (60.5)	3,159 (50.8)	12,045 (70.8)	4,979 (61.5)
NSAIDs	625 (5.0)	212 (3.1)	838 (6.5)	252 (4.1)	1,435 (8.4)	486 (6.0)
Corticosteroids	420 (3.3)	241 (3.5)	866 (6.8)	422 (6.8)	3,081 (18.1)	1,474 (18.2)
SSRI	424 (3.4)	263 (3.8)	720 (5.6)	409 (6.6)	1,971 (11.6)	1,121 (13.9)
CYP3A4/P-gp inhibitors	884 (7.0)	368 (5.4)	1,260 (9.8)	463 (7.5)	2,806 (16.5)	1,063 (13.1)
CYP3A4/P-gp inducers	32 (0.3)	14 (0.2)	47 (0.4)	21 (0.3)	159 (0.9)	65 (0.8)
Proton pump inhibitors	2,521 (20.0)	1,478 (21.5)	4,392 (34.3)	2,370 (38.1)	9,376 (55.1)	4,715 (58.3)
** Other cardiovascular drugs a**						
Beta blocking agents	5,798 (46.0)	2,596 (37.8)	6,946 (54.3)	2,963 (47.7)	9,270 (54.5)	4,001 (49.5)
Diuretics	2,873 (22.8)	1,240 (18.0)	5,434 (42.5)	2,168 (34.9)	10,025 (58.9)	4,090 (50.6)
ACE inhibitors/ARB	4,096 (32.5)	2,021 (29.4)	7,155 (55.9)	3,247 (52.3)	11,437 (67.2)	4,857 (60.0)
Calcium channel blockers	2,864 (22.7)	1,582 (23.0)	4,764 (37.2)	2,197 (35.4)	7,404 (43.5)	3,278 (40.5)
Digoxin	772 (6.1)	192 (2.8)	1,241 (9.7)	345 (5.6)	2,463 (14.5)	755 (9.3)
Statins	3,858 (30.6)	2,160 (31.4)	6,754 (52.8)	3,315 (53.3)	11,367 (66.8)	5,164 (63.8

Abbreviations: ACE, angiotensin converting enzyme; ARB, angiotensin II receptor blockers; CYP, cytochrome P450; DOAC, direct oral anticoagulant; IQR, interquartile range; NSAID, nonsteroidal anti-inflammatory drugs; OAC, oral anticoagulant; P-gp, P-glycoprotein; SSRI, selective serotonin reuptake inhibitors; TIA, transient ischemic attack; VTE, venous thromboembolism.

Note: All values are expressed as n (%), unless otherwise specified.

a Other (i.e., non cardiovascular) drug classes not shown.

In both DOAC and VKA users, median age was 76 years (interquartile range [IQR] 68–82) and the median number of concomitant drugs prescribed was seven (IQR 5–10). The prevalence of comorbidities increased among patients using more concomitant drugs and was similar for VKA and DOAC patients, although heart failure and coronary artery disease were more prevalent in patients using a VKA than in DOAC-users at baseline (unstratified proportions 13.5 vs. 11.7% and 26.3 vs. 23.6%, respectively). Consequently, β blocking agents, diuretics, angiotensin converting enzyme inhibitors/ angiotensin II receptor blockers and digoxin were more often used in the VKA group. In the 6 months prior to the index date, more VKA patients used antiplatelet therapy than DOAC patients. In the first treatment period after the index date however, most antiplatelet drugs had been discontinued but still more VKA patients used concomitant antiplatelet therapy compared with DOAC patients (23.4 vs. 12.7%). The unstratified baseline characteristics for DOAC and VKA users and the baseline characteristics per DOAC per stratum are shown in the Supplementary Material, Section 4 ( Tables A1 and A2 ).

### Primary Outcome

**Fig. 3:**
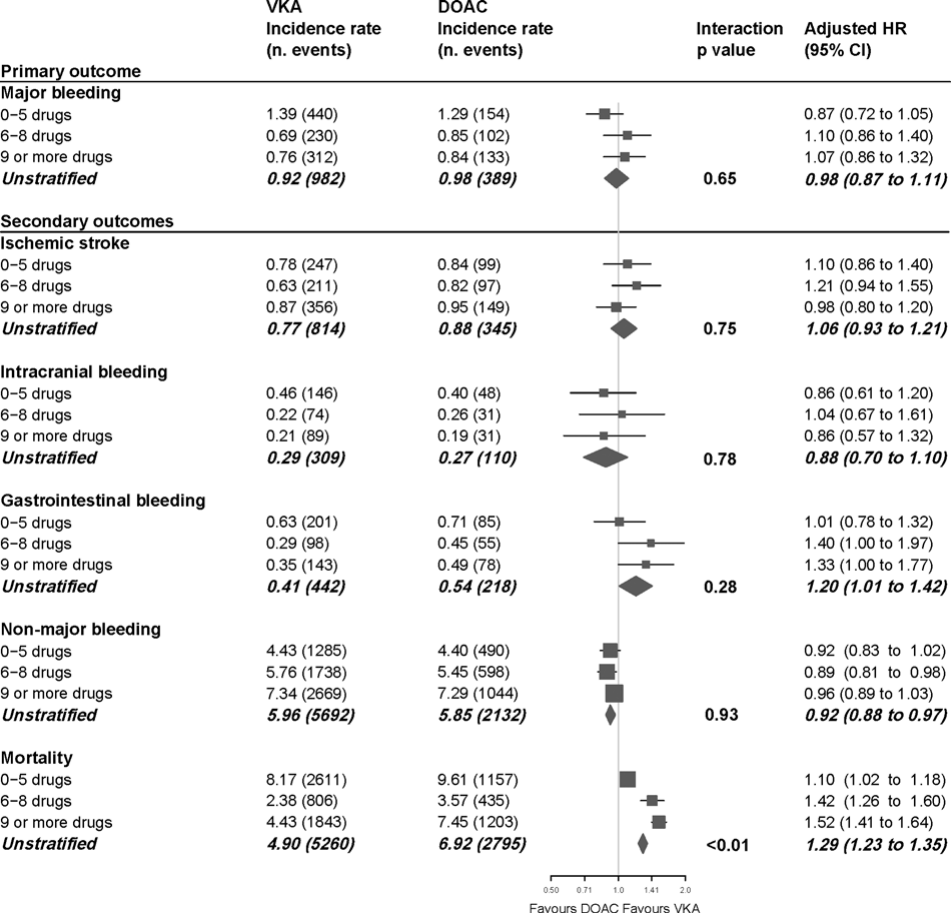
Incidence rates per 100 person-years, interaction *p* -values and adjusted hazard ratios with 95% confidence intervals for primary and secondary outcomes.

The incidence rate of major bleeding was 0.92 per 100 person-years in VKA users, and 0.98 in DOAC users. This was 0.84, 1.12, and 0.83 per 100 person-years for apixaban, rivaroxaban, and dabigatran users, respectively. Fig. 3 shows the incidence rates stratified to the number of concomitant drugs. For both DOAC and VKA users, the incidence rate for major bleeding was highest in the stratum of 0 to 5 concomitant drugs. The adjusted HRs differed slightly among the strata but did not show a clear trend toward a benefit or harm of DOACs versus VKA with an increasing (or decreasing) number of concomitant drugs prescribed. Likewise, the *p* for interaction was not statistically significant ( *p* = 0.65), indicating no effect modification by the number of concomitant drugs prescribed. When comparing DOAC use to VKA use in the unstratified analysis, the crude HR for major bleeding was 1.02 (95% CI 0.91–1.15). After adjustment for all confounders, the HR changed only marginally and indicated no difference in major bleeding risk between DOACs and VKA (adjusted HR 0.98; 95% CI 0.87–1.11).

### Secondary Outcomes

Of the secondary outcomes, effect modification by the number of concomitant drugs prescribed was observed only for the outcome all-cause mortality, with an adjusted HR of 1.52 (95% CI 1.41–1.64) with DOAC use versus VKA use in the highest stratum, compared with an adjusted HR of 1.10 (95% CI 1.02–1.18) in the lowest stratum ( *p* for interaction <0.01). Overall, the mortality rate was almost 30% higher among DOAC users compared with VKA users (unstratified adjusted HR 1.29; 95% CI 1.23–1.35).

For the other outcomes, the number of concomitant drugs prescribed did not modify the effect of DOACs versus VKA ( *p* -values for interaction varied between 0.28 and 0.93). However, in addition to the increased mortality risk, the unstratified analyses also showed an increased risk of gastrointestinal bleeding (adjusted HR 1.20; 95% CI 1.01–1.42) with DOAC use vs. VKA use. Interestingly, in this real-world data we did not observe a reduction with DOAC use compared with VKA use for intracranial bleeding, both in the unstratified and stratified analyses (adjusted HR [unstratified)\] 0.88; 95% CI 0.70–1.10). Also, for ischemic stroke and nonmajor bleeding, no major differences were seen when comparing DOAC and VKA use (see Fig. 3 for details).

The stratified and unstratified results comparing rivaroxaban, apixaban, and dabigatran to VKA separately for the primary outcome major bleeding, are shown in Table 2 . Results for edoxaban are not shown, as the exposure time and the numbers of events were too small to provide reliable results. For all three DOACs, no effect modification by the number of concomitant drugs prescribed was observed for major bleeding ( *p* for interaction 0.67 for apixaban, 0.89 for rivaroxaban, and 0.13 for dabigatran). The unstratified results revealed a statistically significant reduction of major bleeding risk with apixaban only. When comparing the three different DOACs separately to VKA for the outcome mortality, statistically significant effect modification was observed for all three DOACs. The observed overall increased mortality risk with DOACs was not driven by one of the DOACs in particular, as we observed similar increased mortality risks for the three different DOACs when compared with VKA (data not shown). For the outcomes ischemic stroke, gastrointestinal bleeding and intracranial bleeding, the limited number of events did not allow for further stratification comparing the different DOACs to VKA.

**Table 2:** Results of the primary outcome major bleeding for the different DOACs compared with VKA, stratified by number of concomitant drugs

	VKA		Apixaban			Rivaroxaban			Dabigatran		
	Incidence rate per 100 py ( *n* events)	Adjusted HR (95% CI)	Incidence rate per 100 py ( *n* events)	Adjusted HR (95% CI)	Interaction *p* -value	Incidence rate per 100 py ( *n* events)	Adjusted HR (95% CI)	Interaction *p* -value	Incidence rate per 100 py ( *n* events)	Adjusted HR (95% CI)	Interaction *p* -value
Unstratified	0.92 (982)	Ref	0.84 (128)	0.81 (0.68–0.98)	0.67	1.12 (212)	1.15 (0.99–1.34)	0.89	0.83 (44)	0.89 (0.66–1.21)	0.13
0–5	1.39 (440)	Ref	1.01 (43)	0.67 (0.49–0.92)	n.a.	1.59 (93)	1.09 (0.87–1.37)	n.a.	0.94 (16)	0.68 (0.42–1.13)	n.a.
6–8	0.69 (230)	Ref	0.78 (35)	0.96 (0.67–1.38)	n.a.	0.93 (54)	1.26 (0.93–1.70)	n.a.	0.67 (11)	0.99 (0.54–1.80)	n.a.
9 or more	0.76 (312)	Ref	0.78 (50)	0.97 (0.72–1.32)	n.a.	0.89 (65)	1.15 (0.88–1.51)	n.a.	0.86 (17)	1.18 (0.72–1.91)	n.a.

Abbreviations: CI, confidence interval; HR, hazard ratio; n.a., not applicable; Py, person-years; Ref, reference.

### Sensitivity Analyses

Results of the first sensitivity analysis, in which the first period after apparent discontinuation of the anticoagulant was not reclassified to being exposed to the last anticoagulant used, are shown in the Supplementary Material, Section 5 ( Table A3 ). In agreement with the main analysis, no significant effect modification by the number of concomitant drugs prescribed was observed. The unstratified adjusted HR of 1.14 (95% CI 1.01–1.30) showed an increased risk of major bleeding with DOACs compared with VKA, whereas no difference was observed in our main analysis.

Absolute and relative effects in the second, third, and fourth sensitivity analyses were very similar to our main analyses and again did not show signs of effect modification by the number of concomitant drugs on major bleeding (data not shown).

## Discussion

This large, population-based cohort study yielded four principal findings. First, no effect modification by the number of concomitant drugs was observed for the risk of major bleeding when comparing DOACs to VKAs, suggesting that major bleeding risk is comparable between DOACs and VKAs irrespective of the number of concomitant drugs prescribed. Second, we encountered an unexplained higher rate of mortality for DOAC-treated individuals versus VKA-treated individuals which was more pronounced with increasing number of concomitant drugs. Third, in this dataset we did not observe a reduction of intracranial bleeding risk with DOACs compared with VKA, while the risk of gastrointestinal bleeding was increased. Finally, of the individual DOACs, only apixaban significantly reduced major bleeding risk compared with VKA.

### Strengths and Limitations

The main strength of this study is the large size and richness of the data in the United Kingdom CPRD, allowing for thorough adjustment for multiple confounders and stratified analyses. Furthermore, we assessed the data in a time-varying manner, which better reflects the real-life situation in which patients discontinue, start or switch drugs, or develop important comorbidities during follow-up, instead of assuming all variables to remain unchanged throughout follow-up. This also allowed us to identify and address exposure misclassification.

Nevertheless, the high validity of CPRD data notwithstanding, 14 15 an important limitation is that misclassification can still be present. Potential outcome misclassification includes a delayed registration of bleeding events in CPRD after the anticoagulant was stopped because of the bleeding. However, McDonald et al previously showed that the under-recording did not lead to bias. 25 Furthermore, the presence of unmeasured confounding should be considered, although the impact of such residual confounding might be limited as our extensive adjustment for a total of 17 potential confounders did not substantially change the hazard ratios. Another limitation is that we had to rely on Read codes and had no additional information on for instance HbA1c levels or echocardiographic parameters, thereby preventing us from exploring different levels of disease severity. Likewise, we did not have data on causes of death, hospital admissions, blood transfusion, or hemoglobin levels, so the definition of major bleeding was hard to match with definitions used in other studies, notably randomized trials. 18 Also, given the small number of certain events in certain strata of the number of concomitant drugs, our study was not designed nor powered to perform more subgroup analyses. Although of clinical relevance, it is yet unknown if inferences would be different across different patient subgroups such as those with previous TIA/stroke, coronary artery disease, or heart failure. This might be subject to future research. Last, the follow-up duration was relatively short, limiting the power for outcomes that occur infrequently such as ischemic stroke.

### Comparison with Existing Literature

Two smaller propensity score matched cohort studies, using routine care data from the United States, have also investigated whether the number of concomitant drugs modifies bleeding risk of DOACs compared with VKAs. 26 27 Martinez and colleagues also did not find differences in the occurrence of major bleeding between two polypharmacy cohorts comparing rivaroxaban to warfarin. 26 Also in agreement with our results was the study by Mentias and colleagues, who observed a benefit on bleeding risk of apixaban compared with warfarin *only* in the low polypharmacy group, and no differences between apixaban, rivaroxaban, and warfarin in the moderate and high polypharmacy groups. 27

Further research solely consists of post-hoc analyses of the ARISTOTLE trial that showed a statistically significant effect modification ( *p* for interaction 0.017) comparing apixaban with VKA in strata of 0 to 5, 6 to 8 and ≥9 concomitant drugs, 7 and the ROCKET-AF trial that observed a reduction in major bleeding risk in the stratum of 0 to 4 concomitant drugs, but an *increased* risk or inconclusive difference in the higher strata. 8 The results of these two studies were pooled in a systematic review to show significant effect modification by polypharmacy, in which the benefit of DOACs versus VKA on major bleeding disappeared in patients with polypharmacy (pooled relative risk 0.59; 95% CI 0.45–0.76 with <5 drugs, vs. 0.95; 95% CI 0.65–1.39 with ≥5 drugs). 9 This review did not find signs of effect modification by polypharmacy for the outcome stroke or systemic thromboembolism, intracranial bleeding and—in discordance with our results—neither for mortality.

The discrepancies between our study and the RCTs on the effect modification of polypharmacy warrant several considerations. Differences in outcome definitions and, more importantly, patient selection likely account for the fact that the number of concomitant drugs modified the effect of DOACs versus VKAs on major bleeding in trial data, but not in our observational study. Nowadays, both the relatively fit and the frail AF patients in primary and secondary care receive DOAC treatment, whereas patients included in the DOAC trials were likely to be more homogeneous and less frail. Studies show that less than 50% of routine care AF patients would have met the strict inclusion criteria of the DOAC trials. 28 29 Those patients in the highest stratum of the number of concomitant drugs prescribed are therefore more likely to be optimally treated for their many comorbidities, whereas the highest stratum in our study could also include patients who receive too many drugs, including contraindicated or interacting drugs. Likewise, patients in the lowest stratum in the trials probably have little comorbidity and do not need more medication, whereas patients in the lowest stratum in our study can very well be undertreated. On the other hand, although we observed no effect modification, an interesting similar observation in our study and in the previously mentioned observational studies and RCTs is that a benefit of DOACs compared with VKAs regarding major bleeding appears to be absent in patients with polypharmacy.

The finding that the number of concomitant drugs modified mortality risk is more difficult to clarify as we did not have information on the causes of death. Definite conclusions cannot be drawn, though a possible explanation could be residual confounding by (contra-)indication, in which clinicians prefer a DOAC over a VKA especially in patients with many concomitant drugs (and multiple possible interactions that would enhance fluctuating INR levels with VKA use) and that these patients have the highest mortality risk.

In addition, our observation of higher incidence rates among patients receiving fewer concomitant drugs requires further exploration. One explanation may involve issues like end-of-life discontinuation, though this was considered beyond the scope of the current study.

### Clinical Implications and Suggestions for Further Research

In our routine care study population, we did not observe effect modification by the number of concomitant drugs prescribed on major bleeding risk. In practice, this would implicate that clinicians would not need to use the number of concomitant drugs as a tool in deciding which anticoagulant to prescribe in view of major bleeding risk. This study also supports the findings of previous studies that there is no clear benefit on major bleeding of DOACs compared with VKAs in AF patients with polypharmacy, making DOACs an equivalent but not necessarily preferable or superior alternative to VKAs in these patients. 9 26 27

Further research in different routine care datasets or pragmatic trials is needed to confirm our findings in which also the cause of death may be an important outcome to consider. Furthermore, as our study aimed to investigate the influence of the total number of concomitant drugs used, we did not study different drug classes. Individual (interacting) drugs may certainly influence the (comparative) risk of bleeding (e.g., antiplatelet use). Future research may focus specifically on pharmacokinetic and dynamic interactions as part of concomitant drug use in DOAC treatment. Last, although one could regard the number of concomitant drugs prescribed as a proxy for frailty, it remains uncertain whether DOACs are completely safe in frail elderly AF patients. A high number of concomitant drugs could also indicate that someone is adequately treated and not necessarily frail. Therefore, studies comparing DOACs to VKA in frail patients, like the FRAIL-AF trial, will have to be awaited before this question can be answered. 30

## Conclusion

Major bleeding risk was comparable between DOACs and VKAs, irrespective of the number of concomitant drugs prescribed. Further research including an assessment of the causes of death is required before drawing conclusions on possible increased mortality risk with DOACs and effect modification of the number of concomitant drugs concerning mortality.
