# Supplementary material for: The Number of Concomitant Drugs and the Safety of Direct Oral Anticoagulants in Routine Care Patients with Atrial Fibrillation
Source: TH Open. 2020 Dec 23;4(4):e417–26. doi: 10.1055/s-0040-1721499 (PMC7758151; doi:10.1055/s-0040-1721499)
Supplement: Supplementary file 1 — Supplementary Material [file 10-1055-s-0040-1721499-s200079.pdf]

Supplementary Material

1. Read Codes Defining Outcomes

Major bleeding (including intracerebral and gastrointestinal bleeding)
All codes up to G621.00 were used for the outcome intracranial bleeding
All codes from G850.00 up to G852000 were used for the outcome gastrointestinal bleeding

readcode	Readterm
S62..00	Cerebral hemorrhage following injury
S62..11	Extradural hemorrhage following injury
S62..12	Subarachnoid hemorrhage following injury
S62..13	Subdural hemorrhage following injury
S62..14	Traumatic cerebral hemorrhage
S620.00	Closed traumatic subarachnoid hemorrhage
S620100	Subarachnoid hemorrhage injury no open intracranial wound + no loss concussion
S620600	Subarachnoid hemorrhage injury no open intracranial wound + LOC unspecified duration
S620z00	Subarachnoid hemorrhage injury no open intracranial wound + concussion unspecified
S621.00	Open traumatic subarachnoid hemorrhage
S621z00	Subarachnoid hemorrhage injury + open intracranial wound + concussion unspecified
S622.00	Closed traumatic subdural hemorrhage
S622000	Subdural hemorrhage injury no open intracranial wound + unspecified concussion
S622300	Subdural hemorrhage injury no open intracranial wound + 1–24 h loss concussion
S622600	Subdural hemorrhage injury no open intracranial wound + LOC unspecified duration
S622z00	Subdural hemorrhage injury no open intracranial wound + concussion unspecified
S623.00	Open traumatic subdural hemorrhage
S624.00	Closed traumatic extradural hemorrhage
S624000	Extradural hemorrhage injury no open intracranial wound + unspecified concussion
S624100	Extradural hemorrhage injury no open intracranial wound + no loss concussion
S624z00	Extradural hemorrhage injury no open intracranial wound + concussion unspecified
S625.00	Open traumatic extradural hemorrhage
S626.00	Epidural hemorrhage
S627.00	Traumatic subarachnoid hemorrhage
S628.00	Traumatic subdural hemorrhage
S62z.00	Cerebral hemorrhage following injury NOS
S63..00	Other cerebral hemorrhage following injury
S630.00	Other cerebral hemorrhage after injury no open intracranial wound
S630.12	Intracranial hematoma following injury

(*Continued*)

(*Continued*)

readcode	Readterm
S630000	Other cerebral hemorrhage injury no open intracranial wound + unspecified concussion
S630100	Other cerebral hemorrhage injury no open intracranial wound + no loss concussion
S630200	Other cerebral hemorrhage injury no open intracranial wound + < 1 h loss concussion
S630300	Other cerebral hemorrhage injury no open intracranial wound + 1–24 h LOC
S630400	Other cerebral hemorrhage injury no open intracranial wound + > 24 h LOC +recovery
S631300	Other cerebral hemorrhage injury + open intracranial wound + 1–24 h loss concussion
S63z.00	Other cerebral hemorrhage following injury NOS
G60..00	Subarachnoid hemorrhage
G600.00	Ruptured berry aneurysm
G601.00	Subarachnoid hemorrhage from carotid siphon and bifurcation
G602.00	Subarachnoid hemorrhage from middle cerebral artery
G603.00	Subarachnoid hemorrhage from anterior communicating artery
G604.00	Subarachnoid hemorrhage from posterior communicating artery
G605.00	Subarachnoid hemorrhage from basilar artery
G606.00	Subarachnoid hemorrhage from vertebral artery
G60X.00	Subarachnoid hemorrhage from intracranial artery, unspecified
G60z.00	Subarachnoid hemorrhage NOS
G61..00	Intracerebral hemorrhage
G61..11	CVA—cerebrovascular accident due to intracerebral hemorrhage
G61..12	Stroke due to intracerebral hemorrhage
G610.00	Cortical hemorrhage
G611.00	Internal capsule hemorrhage
G612.00	Basal nucleus hemorrhage
G613.00	Cerebellar hemorrhage
G614.00	Pontine hemorrhage
G615.00	Bulbar hemorrhage
G616.00	External capsule hemorrhage
G617.00	Intracerebral hemorrhage, intraventricular

(*Continued*)

(*Continued*)

readcode	Readterm
G618.00	Intracerebral hemorrhage, multiple localized
G619.00	Lobar cerebral hemorrhage
G61X.00	Intracerebral hemorrhage in hemisphere, unspecified
G61 × 000	Left-sided intracerebral hemorrhage, unspecified
G61 × 100	Right-sided intracerebral hemorrhage, unspecified
G61z.00	Intracerebral hemorrhage NOS
G62..00	Other and unspecified intracranial hemorrhage
Gyu6000	[X]Subarachnoid hemorrhage from other intracranial arteries
Gyu6100	[X]Other subarachnoid hemorrhage
Gyu6200	[X]Other intracerebral hemorrhage
Gyu6E00	[X]Subarachnoid hemorrhage from intracranial artery, unspecified
Gyu6F00	[X]Intracerebral hemorrhage in hemisphere, unspecified
G62z.00	Intracranial hemorrhage NOS
G622.00	Subdural hematoma—nontraumatic
G623.00	Subdural hemorrhage NOS
G621.00	Subdural hemorrhage—nontraumatic
1720.00	Massive hemoptysis
D211.00	Acute post hemorrhagic anemia
D211.11	Normocytic anemia following acute bleed
F212.00	Acute and subacute hemorrhagic leukoence-phalitis [Hurst]
F404300	Hemophthalmos (excluding current injury)
F404500	Intraocular hemorrhage
F42y.11	Hemorrhage—retinal
F42y000	Preretinal hemorrhage
F42y100	Superficial retinal hemorrhage
F42y300	Deep retinal hemorrhage
F42y400	Subretinal hemorrhage
F42y500	Retinal hemorrhage NOS
F436.00	Choroidal hemorrhage and rupture
F436000	Unspecified choroidal hemorrhage
F436100	Expulsive choroidal hemorrhage
F436z00	Choroidal hemorrhage or rupture NOS
F437200	Hemorrhagic choroidal detachment
F4K2800	Vitreous hemorrhage
FyuH400	[X]Vitreous hemorrhage in diseases classified elsewhere
G8y0.00	Hemorrhage NOS
H51y200	Hemothorax
N091.00	Hemarthrosis
N091000	Hemarthrosis of unspecified site

(*Continued*)

readcode	Readterm
G850.00	Esophageal varices with bleeding
J10y000	Hemorrhage of esophagus
J68..00	Gastrointestinal hemorrhage
J68z.00	Gastrointestinal hemorrhage unspecified
J68z.11	GIB—gastrointestinal bleeding
J68z200	Upper gastrointestinal hemorrhage
J68zz00	Gastrointestinal tract hemorrhage NOS
J68z000	Gastric hemorrhage NOS
J68z100	Intestinal hemorrhage NOS
J681.00	Melaena
J680.00	Hematemesis
J680.11	Vomiting of blood
J110100	Acute gastric ulcer with hemorrhage
J110300	Acute gastric ulcer with hemorrhage and perforation
J111100	Chronic gastric ulcer with hemorrhage
J111300	Chronic gastric ulcer with hemorrhage and perforation
J11y100	Unspecified gastric ulcer with hemorrhage
J11yy00	Unspecified gastric ulcer; unspecified hemor-rhage and/or perforation
J120100	Acute duodenal ulcer with hemorrhage
J120300	Acute duodenal ulcer with hemorrhage and perforation
J121100	Chronic duodenal ulcer with hemorrhage
J121300	Chronic duodenal ulcer with hemorrhage and perforation
J12y100	Unspecified duodenal ulcer with hemorrhage
J12y300	Unspecified duodenal ulcer with hemorrhage and perforation
J12yy00	Unspecified duodenal ulcer; unspecified hem-orrhage and/or perforation
J130100	Acute peptic ulcer with hemorrhage
J13y100	Unspecified peptic ulcer with hemorrhage
J131100	Chronic peptic ulcer with hemorrhage
J130300	Acute peptic ulcer with hemorrhage and perforation
J13y300	Unspecified peptic ulcer with hemorrhage and perforation
J140100	Acute gastrojejunal ulcer with hemorrhage
J140300	Acute gastrojejunal ulcer with hemorrhage and perforation
J141300	Chronic gastrojejunal ulcer with hemorrhage and perforation
J14y100	Unspecified gastrojejunal ulcer with hemorrhage
J150000	Acute hemorrhagic gastritis
J56y000	Hemoperitoneum—nontraumatic
J121111	Bleeding chronic duodenal ulcer

(*Continued*)

readcode	Readterm
J111111	Bleeding chronic gastric ulcer
J110111	Bleeding acute gastric ulcer
G852000	Esophageal varices with bleeding in diseases EC
Ischemic stroke	
G63..11	Infarction—precerebral
G64..00	Cerebral arterial occlusion
G64..11	CVA—cerebral artery occlusion
G64..12	Infarction—cerebral
G64..13	Stroke due to cerebral arterial occlusion
G640.00	Cerebral thrombosis
G640000	Cerebral infarction due to thrombosis of cerebral arteries
G641.00	Cerebral embolism
G641.11	Cerebral embolus
G641000	Cerebral infarction due to embolism of cerebral arteries
G64z.00	Cerebral infarction NOS
G64z.11	Brainstem infarction NOS
G64z.12	Cerebellar infarction
G64z000	Brainstem infarction
G64z100	Wallenberg syndrome
G64z111	Lateral medullary syndrome
G64z200	Left-sided cerebral infarction
G64z300	Right-sided cerebral infarction
G65..13	Vertebrobasilar insufficiency
G650.00	Basilar artery syndrome
G650.11	Insufficiency—basilar artery
G651.00	Vertebral artery syndrome
G651000	Vertebrobasilar artery syndrome
G66..00	Stroke and cerebrovascular accident unspecified
G66..11	CVA unspecified
G66..12	Stroke unspecified
G66..13	CVA—cerebrovascular accident unspecified
G660.00	Middle cerebral artery syndrome
G661.00	Anterior cerebral artery syndrome
G662.00	Posterior cerebral artery syndrome
G663.00	Brainstem stroke syndrome
G664.00	Cerebellar stroke syndrome
G665.00	Pure motor lacunar syndrome
G666.00	Pure sensory lacunar syndrome
G667.00	Left-sided CVA
G668.00	Right-sided CVA
G676000	Cerebral infarct due to cerebral venous thrombosis, nonpyogenic
Gyu6300	[X]Cerebral infarction due/unspecified occlusion or stenosis/cerebral arteries

(*Continued*)

(*Continued*)

readcode	Readterm
Gyu6400	[X]Other cerebral infarction
Gyu6E00	[X]Subarachnoid hemorrhage from intracranial artery, unspecified
Gyu6G00	[X]Cerebral infarct due unspecified occlusion/stenosis precerebral arteries
G654.00	Multiple and bilateral precerebral artery syndromes
G64z400	Infarction of basal ganglia
Nonmajor bleeding	
172..00	Blood in sputum—hemoptysis
172..12	Hemoptysis—symptom
1A45.00	Blood in urine—hematuria
1A45.12	Hematuria—symptom
1C6..11	Epistaxis symptom
1C62.00	Has nose bleeds—epistaxis
2D25.00	O/E—epistaxis
F4C7100	Subconjunctival hemorrhage
F4C7200	Conjunctival hemorrhage NOS
F4G3200	Exophthalmos due to orbital hemorrhage
F4K7.00	Retrobulbar hemorrhage
K0A2.00	Recurrent and persistent hematuria
K0A2000	Recurrent + persistent hematuria minor glomerular abnormality
K0A2100	Recurrent + persistent hematuria, focal + segmental glomerular lesions
K0A2200	Recurrent + persist hematuria diffuse membranous glomerulonephritis
K0A2600	Recurrent and persistent hematuria, dense deposit disease
K197.00	Hematuria
K197.12	Essential hematuria
K197000	Painless hematuria
K197100	Painful hematuria
K197300	Frank hematuria
K197400	Clot hematuria
K5A1.00	Postmenopausal bleeding
R047.00	[D]Epistaxis
R048.00	[D]Throat hemorrhage
R063.00	[D]Hemoptysis
R063z00	[D]Hemoptysis NOS
K167.00	Hemorrhage into bladder wall
J681.11	Blood in stool
J681.13	Blood in stools altered
J681.12	Altered blood in stools
J573.00	Hemorrhage of rectum and anus
J573000	Rectal hemorrhage
J573100	Anal hemorrhage

(*Continued*)

(*Continued*)

readcode	Readterm
J573z00	Hemorrhage of rectum and anus NOS
J573011	Rectal bleeding
J573.11	Bleeding PR
J510900	Bleeding diverticulosis
J573012	PRB—rectal bleeding

Abbreviations: CVA, cerebrovascular accident; NOS, nitric oxide synthase; PR, per rectal; PRB, per rectal bleed.

2. Confounders Per Outcome

Ischemic Stroke (16 Confounders)

Age, sex, previous use of a different anticoagulant, alcohol abuse, liver disease, chronic kidney disease, hypertension, hypercholesterolemia (including statin use), ischemic heart disease or peripheral artery disease, history of stroke or TIA, history of venous thromboembolism, active cancer, congestive heart failure, chronic kidney disease, diabetes, concomitant use of platelet inhibitors.

Intracranial Bleeding (11 Confounders)

Age, sex, previous use of a different anticoagulant, alcohol abuse, liver disease, chronic kidney disease, hypertension, history of intracranial bleeding, history of gastrointestinal bleeding, cardiovascular disease (defined as a history of ischemic heart disease, peripheral artery disease, stroke or transient ischemic attack [TIA]), concomitant use of platelet inhibitors.

Gastrointestinal Bleeding (17 Confounders, Same As for Major Bleeding)

Age, sex, previous use of a different anticoagulant, alcohol abuse, liver disease, chronic kidney disease, hypertension, history of gastrointestinal bleeding, history of intracranial bleeding, cardiovascular disease (defined as a history of ischemic heart disease, peripheral artery disease, stroke or TIA), active cancer, peptic ulcer disease, concomitant use of platelet inhibitors, nonsteroidal anti-inflammatory drugs (NSAIDs), oral corticosteroids, proton pump inhibitors (PPIs), or selective serotonin reuptake inhibitors (SSRIs).

CRNMB (18 Confounders, Including History of CRNMB)

Age, sex, previous use of a different anticoagulant, alcohol abuse, liver disease, chronic kidney disease, hypertension, history of CRNMB, history of gastrointestinal bleeding, history of intracranial bleeding, cardiovascular disease (defined as a history of ischemic heart disease, peripheral artery disease, stroke, or TIA), active cancer, peptic ulcer disease, concomitant use of platelet inhibitors, NSAIDs, oral corticosteroids, PPIs, or SSRIs.

Death (21 Confounders)

Age, sex, previous use of a different anticoagulant, alcohol abuse, liver disease, chronic kidney disease, hypertension, hypercholesterolemia (including statin use), congestive heart failure, history of gastrointestinal bleeding, history of intracranial bleeding, cardiovascular disease (defined as a history of ischemic heart disease, peripheral artery disease, stroke or TIA), history of venous thromboembolism, active cancer, peptic ulcer disease, diabetes, concomitant use of platelet inhibitors, NSAIDs, oral corticosteroids, PPIs, or SSRIs.

3. BNF Codes Left Out of Number of Concomitant Drugs Variable for Third Sensitivity Analyses

List of BNF chapters of topical drugs

1.7.4: Management of Anal Fissures
11: Eye
11.3: Anti-Infective Eye Preparations
11.3.1: Antibacterials
11.3.2: Antifungals
11.3.3: Antivirals
11.4: Corticosteroids & Other Anti-Inflammatory Preparations.
11.4.1: Corticosteroids
11.4.2: Other Anti-Inflammatory Preparations
11.5: Mydriatics And Cycloplegics
11.6: Treatment of Glaucoma
11.7: Local Anaesthetics
11.8: Miscellaneous Ophthalmic Preparations
11.8.1: Tear Deficiency, Eye Lubricant/Astringent
11.8.2: Ocular Diagnosis/Peri-op Preparation & Photodyn Tt
11.8.3: Other Eye Preparations
12: Ear, Nose and Oropharynx
12.1: Drugs Acting on The Ear
12.1.1: Otitis Externa
12.1.3: Removal of Ear Wax & Other Substances
12.2: Drugs Acting on The Nose
12.2.1: Drugs Used in Nasal Allergy
12.2.2: Topical Nasal Decongestants
12.2.3: Nasal Preparation for Infection
12.3: Drugs Acting on The Oropharynx
12.3.1: Drugs for Oral Ulceration & Inflammation
12.3.2: Oropharyngeal Anti-Infective Drugs
12.3.3: Lozenges & Sprays
12.3.4: Mouthwashes, Gargles, And Dentifrices
12.3.5: Treatment of Dry Mouth
13: Skin
13.1: Management of Skin Conditions
13.2: Emollient & Barrier Preparations
13.3: Top Local Anaesthetics & Antipruritics
13.4: Topical Corticosteroids
13.5: Preparations for Eczema and Psoriasis
13.6: Acne and Rosacea
13.7: Preparations for Warts and Calluses

13.10: Anti-Infective Skin Preparations
13.14: Topical Circulatory Preparations
13.15: Miscellaneous Topical Preparations
21: Appliances
21.14: Lubricant Gels
21.16: Irrigation Solutions
21.21: Dry Mouth Products
21.22: Emollients
21.23: Vaginal Moisturizers
21.24: Nasal Products
21.34: Vaginal PH Correction Products
List of BNF chapters of incidental drugs
2.8.1: Parenteral Anticoagulants
4.8.2: Drugs Used in Status Epilepticus
7.3.3: Spermicidal Contraceptives
7.3.5: Emergency Contraception
9.2.2: Parent Preparation for Fluid & Electrolyte Imb
9.3: Intravenous Nutrition
14.3: Diagnostic Vaccines
14.4: Vaccines and Antisera
15.1: General Anesthesia
19.2.7: Poisoning Antidotes
21.43: Micro-Enema—Sodium Citrate

4. Unstratified Baseline Characteristics

Table A1 Baseline characteristics, unstratified

	VKA ($n = 42,424$)	DOAC ($n = 21,176$)
Female	18,573 (43.8)	9,438 (44.6)
Age, median (IQR)	76 (68–82)	76 (68–83)
Number of concomitant drugs prescribed, median (IQR)	7 (5–10)	7 (5–10)
Previous use of different OAC	5 (0.0)	1,122 (5.3)
Comorbidities/risk factors		
Hypertension	26,995 (63.6)	13,158 (62.1)
Congestive heart failure	5,726 (13.5)	2,488 (11.7)
Diabetes	7,789 (18.4)	3,962 (18.7)
Prior TIA or ischemic stroke	7,621 (18.0)	4,070 (19.2)
Prior venous thromboembolism	1,996 (4.7)	631 (3.0)
Coronary artery disease	11,164 (26.3)	5,002 (23.6)
Presence of malignancy	1,559 (3.7)	795 (3.8)
Chronic kidney disease	9,992 (23.6)	4,624 (21.8)
Prior major bleeding	1,975 (4.7)	1,073 (5.1)
Peptic ulcer disease	2,773 (6.5)	1,410 (6.7)
Alcohol abuse	3,176 (7.5)	2,251 (10.6)
Active smoking	3,585 (8.5)	1,969 (9.3)
Prior use of drugs affecting bleeding risk		
Antiplatelet therapy	25,727 (60.6)	10,479 (49.5)
NSAID	2,898 (6.8)	950 (4.5)
Corticosteroids	4,367 (10.3)	2,137 (10.1)
SSRI	3,115 (7.3)	1,793 (8.5)
CYP3A4 or P-gp inhibitors	4,950 (11.7)	1,894 (8.9)
CYP3A4 or P-gp inducers	238 (0.6)	100 (0.5)
Proton pump inhibitors	16,289 (38.4)	8,563 (40.4)
Other cardiovascular drugs[a]		
Beta blocking agents	22,014 (51.9)	9,560 (45.1)
Diuretics	18,332 (43.2)	7,498 (35.4)
ACE inhibitors/ARB	22,688 (53.5)	10,125 (47.8)
Calcium channel blockers	15,032 (35.4)	7,057 (33.3)
Digoxin	4,476 (10.6)	1,292 (6.1)
Statins	21,979 (51.8)	10,639 (50.2)

Abbreviations: ACE, angiotensin converting enzyme; ARB, angiotensin II receptor blockers; CYP, cytochrome P450; IQR, interquartile range; NSAID, nonsteroidal anti-inflammatory drugs; OAC, oral anticoagulant; P-gp, P-glycoprotein; SSRI, selective serotonin reuptake inhibitors; TIA, transient ischemic attack.
Note: All values are expressed as *n* (%), unless otherwise specified; Active cancer at baseline was defined as having a Read code for any type of cancer in the 6 mo preceding the index date; All other comorbidities/risk factors were considered present when a Read code was registered ever/before the index date; Drugs prescribed in the 6 mo prior to the index date were regarded as used at baseline.
[a]Other (i.e., noncardiovascular) drug classes not shown.

Table A2 Baseline characteristics stratified per DOAC and per number of concomitant drugs stratum

	Apixaban			Dabigatran			Edoxaban			Rivaroxaban			VKA		
No. of concomitant drugs	0–5	6–8	≥9	0–5	6–8	≥9	0–5	6–8	≥9	0–5	6–8	≥9	0–5	6–8	≥9
N	2,685 (100.0)	2,611 (100.0)	3,733 (100.0)	803 (100.0)	666 (100.0)	720 (100.0)	158 (100.0)	106 (100.0)	113 (100.0)	3,226 (100.0)	2,831 (100.0)	3,524 (100.0)	12,607 (100.0)	12,798 (100.0)	17,019 (100.0)
Female	1,079 (40.2)	1,182 (45.3)	1,911 (51.2)	268 (33.4)	248 (37.2)	351 (48.8)	61 (38.6)	45 (42.5)	50 (44.2)	1,231 (38.2)	1,225 (43.3)	1,787 (50.7)	4,715 (37.4)	5,654 (44.2)	8,204 (48.2)
Age, median (IQR)	72.01 (11.91)	76.13 (10.69)	78.11 (9.87)	70.89 (11.48)	74.39 (10.27)	77.40 (9.41)	72.71 (11.83)	75.91 (11.41)	78.04 (8.97)	71.85 (11.90)	75.81 (10.18)	77.68 (9.72)	71.17 (11.61)	74.91 (9.81)	76.14 (9.24)
N. conc. drugs, median (IQR)	4 (3–5)	7 (6–8)	11 (10–14)	4 (3–5)	7 (6–8)	11 (10–13)	4 (2–5)	7 (6–8)	11 (10–13)	4 (3–5)	7 (6–8)	11 (10–14)	4 (3–5)	7 (6–8)	11 (10–14)
Previous use of different OAC	93 (3.5)	100 (3.8)	197 (5.3)	71 (8.8)	62 (9.3)	71 (9.9)	7 (4.4)	3 (2.8)	4 (3.5)	142 (4.4)	151 (5.3)	223 (6.3)	974 (7.7)	1,035 (8.1)	1,566 (9.2)
Comorbidities/risk factors															
Hypertension	1,197 (44.6)	1,725 (66.1)	2,724 (73.0)	352 (43.8)	461 (69.2)	531 (73.8)	80 (50.6)	68 (64.2)	79 (69.9)	1,473 (45.7)	1,912 (67.5)	2,556 (72.5)	5,806 (46.1)	8,604 (67.2)	12,585 (73.9)
Heart failure	136 (5.1)	299 (11.5)	704 (18.9)	46 (5.7)	63 (9.5)	124 (17.2)	18 (11.4)	7 (6.6)	24 (21.2)	165 (5.1)	292 (10.3)	610 (17.3)	813 (6.4)	1,591 (12.4)	3,322 (19.5)
Diabetes	194 (7.2)	411 (15.7)	1,135 (30.4)	41 (5.1)	106 (15.9)	208 (28.9)	13 (8.2)	14 (13.2)	31 (27.4)	227 (7.0)	507 (17.9)	1,075 (30.5)	804 (6.4)	1,833 (14.3)	5,152 (30.3)
Prior TIA or ischemic stroke	426 (15.9)	574 (22.0)	922 (24.7)	131 (16.3)	132 (19.8)	177 (24.6)	14 (8.9)	24 (22.6)	20 (17.7)	386 (12.0)	494 (17.4)	770 (21.9)	1,661 (13.2)	2,413 (18.9)	3,547 (20.8)
Prior VTE	38 (1.4)	55 (2.1)	125 (3.3)	16 (2.0)	18 (2.7)	16 (2.2)	2 (1.3)	1 (0.9)	5 (4.4)	89 (2.8)	89 (3.1)	177 (5.0)	509 (4.0)	534 (4.2)	953 (5.6)
Coronary artery disease	300 (11.2)	609 (23.3)	1,358 (36.4)	84 (10.5)	152 (22.8)	254 (35.3)	18 (11.4)	22 (20.8)	41 (36.3)	331 (10.3)	632 (22.3)	1,201 (34.1)	1,408 (11.2)	3,012 (23.5)	6,744 (39.6)
Presence of malignancy	83 (3.1)	90 (3.4)	138 (3.7)	27 (3.4)	28 (4.2)	28 (3.9)	5 (3.2)	2 (1.9)	5 (4.4)	102 (3.2)	124 (4.4)	163 (4.6)	403 (3.2)	426 (3.3)	730 (4.3)
Chronic kidney disease	309 (11.5)	547 (20.9)	1,183 (31.7)	88 (11.0)	128 (19.2)	198 (27.5)	18 (11.4)	22 (20.8)	31 (27.4)	411 (12.7)	623 (22.0)	1,066 (30.2)	1,662 (13.2)	2,914 (22.8)	5,416 (31.8)
Prior major bleeding	99 (3.7)	155 (5.9)	272 (7.3)	24 (3.0)	41 (6.2)	41 (5.7)	8 (5.1)	3 (2.8)	4 (3.5)	95 (2.9)	103 (3.6)	228 (6.5)	344 (2.7)	548 (4.3)	1,083 (6.4)
Peptic ulcer disease	119 (4.4)	194 (7.4)	358 (9.6)	28 (3.5)	44 (6.6)	64 (8.9)	13 (8.2)	7 (6.6)	8 (7.1)	129 (4.0)	164 (5.8)	282 (8.0)	534 (4.2)	807 (6.3)	1,432 (8.4)
Alcohol abuse	274 (10.2)	248 (9.5)	423 (11.3)	56 (7.0)	72 (10.8)	77 (10.7)	15 (9.5)	23 (21.7)	18 (15.9)	322 (10.0)	320 (11.3)	403 (11.4)	847 (6.7)	942 (7.4)	1,387 (8.1)
Active smoking	238 (8.9)	228 (8.7)	365 (9.8)	80 (10.0)	48 (7.2)	56 (7.8)	12 (7.6)	11 (10.4)	17 (15.0)	275 (8.5)	263 (9.3)	376 (10.7)	1,070 (8.5)	1,040 (8.1)	1,475 (8.7)
Prior use of drugs affecting bleeding risk															
Antiplatelet therapy	855 (31.8)	1,290 (49.4)	2,249 (60.2)	348 (43.3)	398 (59.8)	508 (70.6)	51 (32.3)	47 (44.3)	66 (58.4)	1,087 (33.7)	1,424 (50.3)	2,156 (61.2)	5,937 (47.1)	7,745 (60.5)	12,045 (70.8)
NSAIDS	75 (2.8)	97 (3.7)	216 (5.8)	37 (4.6)	32 (4.8)	54 (7.5)	3 (1.9)	8 (7.5)	6 (5.3)	97 (3.0)	115 (4.1)	210 (6.0)	625 (5.0)	838 (6.5)	1,435 (8.4)
Corticosteroids	98 (3.6)	166 (6.4)	666 (17.8)	24 (3.0)	34 (5.1)	123 (17.1)	8 (5.1)	6 (5.7)	19 (16.8)	111 (3.4)	216 (7.6)	666 (18.9)	420 (3.3)	866 (6.8)	3,081 (18.1)
SSRI	105 (3.9)	175 (6.7)	581 (15.6)	22 (2.7)	30 (4.5)	80 (11.1)	8 (5.1)	7 (6.6)	14 (12.4)	128 (4.0)	197 (7.0)	446 (12.7)	424 (3.4)	720 (5.6)	1,971 (11.6)
CYP3A4/P-gp inhibitors	142 (5.3)	203 (7.8)	471 (12.6)	49 (6.1)	43 (6.5)	103 (14.3)	6 (3.8)	13 (12.3)	7 (6.2)	171 (5.3)	204 (7.2)	482 (13.7)	884 (7.0)	1,260 (9.8)	2,806 (16.5)
CYP3A4/P-gp inducers	3 (0.1)	6 (0.2)	27 (0.7)	2 (0.2)	3 (0.5)	9 (1.2)	0 (0.0)	1 (0.9)	0 (0.0)	9 (0.3)	11 (0.4)	29 (0.8)	32 (0.3)	47 (0.4)	159 (0.9)
Proton pump inhibitors	606 (22.6)	1,025 (39.3)	2,210 (59.2)	143 (17.8)	237 (35.6)	413 (57.4)	39 (24.7)	47 (44.3)	70 (61.9)	690 (21.4)	1,061 (37.5)	2,022 (57.4)	2,521 (20.0)	4,392 (34.3)	9,376 (55.1)
Other cardiovascular drugs[a]															
Beta blocking agents	979 (36.5)	1,232 (47.2)	1,874 (50.2)	357 (44.5)	348 (52.3)	378 (52.5)	54 (34.2)	47 (44.3)	45 (39.8)	1,206 (37.4)	1,336 (47.2)	1,704 (48.4)	5,798 (46.0)	6,946 (54.3)	9,270 (54.5)
Diuretics	501 (18.7)	900 (34.5)	1,859 (49.8)	154 (19.2)	240 (36.0)	388 (53.9)	40 (25.3)	42 (39.6)	55 (48.7)	545 (16.9)	986 (34.8)	1,788 (50.7)	2,873 (22.8)	5,434 (42.5)	10,025 (58.9)
ACE inhibitors/ARB	793 (29.5)	1,343 (51.4)	2,187 (58.6)	226 (28.1)	367 (55.1)	453 (62.9)	52 (32.9)	55 (51.9)	77 (68.1)	950 (29.4)	1,482 (52.3)	2,140 (60.7)	4,096 (32.5)	7,155 (55.9)	11,437 (67.2)
Calcium channel blockers	624 (23.2)	885 (33.9)	1,502 (40.2)	170 (21.2)	236 (35.4)	275 (38.2)	33 (20.9)	35 (33.0)	58 (51.3)	755 (23.4)	1,041 (36.8)	1,443 (40.9)	2,864 (22.7)	4,764 (37.2)	7,404 (43.5)
Digoxin	51 (1.9)	125 (4.8)	299 (8.0)	42 (5.2)	58 (8.7)	107 (14.9)	4 (2.5)	1 (0.9)	2 (1.8)	95 (2.9)	161 (5.7)	347 (9.8)	772 (6.1)	1,241 (9.7)	2,463 (14.5)
Statins	868 (32.3)	1,393 (53.4)	2,414 (64.7)	260 (32.4)	358 (53.8)	445 (61.8)	53 (33.5)	57 (53.8)	78 (69.0)	979 (30.3)	1,507 (53.2)	2,227 (63.2)	3,858 (30.6)	6,754 (52.8)	11,367 (66.8)

Abbreviations: ACE, angiotensin converting enzyme; ARB, angiotensin II receptor blockers; CYP, cytochrome P450; NSAID, nonsteroidal anti-inflammatory drugs; OAC, oral anticoagulant; P-gp, P-glycoprotein; SSRI, selective serotonin reuptake inhibitors; TIA, transient ischemic attack.

[a]Other (i.e., noncardiovascular) drug classes not shown.

Note: All values are expressed as *n* (%), unless otherwise specified. Active cancer at baseline was defined as having a Read code for any type of cancer in the 6 mo preceding the index date. All other comorbidities/risk factors were considered present when a Read code was registered ever/before the index date. Drugs prescribed in the 6 mo prior to the index date were regarded as used at baseline.

5. Sensitivity Analysis Exposure Misclassification

Table A3 Unstratified and stratified results (per stratum of the number of concomitant drugs prescribed) for primary outcome without reclassification of first unexposed period

	VKA ($n = 42{,}424$)	DOAC ($n = 21{,}176$)	DOAC vs. VKA	
	Incidence rate per 100 py (*n* events)	Incidence rate per 100 py (*n* events)	Crude HR (95% CI, *p*)	Adjusted HR[a] (95% CI, *p*)
Major bleeding				
0–5	1.10 (290)	1.19 (131)	1.04 (0.84–1.28, 0.74)	1.06 (0.86–1.31, 0.57)
6–8	0.63 (192)	0.82 (96)	1.23 (0.96–1.57, 0.11)	1.19 (0.92–1.52, 0.18)
9 or more	0.69 (262)	0.82 (128)	1.17 (0.95–1.45, 0.14)	1.18 (0.95–1.46, 0.13)
Unstratified	0.78 (744)	0.93 (355)	1.14 (1.01–1.30, 0.04)	1.14 (1.01–1.30, 0.04)

Abbreviations: HR, hazard ratio; CI, confidence interval.
[a]Multivariate adjusted HR (for the list of confounders, see text).
